# Notes and News

**Published:** 1900-08-04

**Authors:** 


### Notes and News.

The congress of Spanish surgeons, which was to have been held in September, has been postponed until the following year.

Birmingham has been very fortunate recently in the munificence shown to it. Lord Calthorpe has given a site of 25 acres for the new scientific department of the University, and Sir James Chance has made a donation of £50,000 towards the funds of the University.

The report of the Plague Commission in India is likely to cause considerable modification in the measures taken to stay the spread of the disease in native quarters. Districts will be isolated as stringently as ever, and communications with infected places carefully guarded, but there will be less interference in the domiciliary arrangements in respect to native feeling.

Two donations of £1,000 each have been received by St. Thomas's Hospital. One is for the purpose of endowing a "South Bed," in memory of the late Mr. J. F. South, F.R.C.S., formerly surgeon at the hospital, presented by his two daughters, and the other is a legacy to endow the "Matthew and Patience Goddard Bed," bequeathed by Mr. A. M. Goddard.

The Council of the Society for the Study of Disease in Children has been formed, and consists of Dr. Ashby, Manchester; Mr. H. Armstrong, Wellington College; Dr. Fletcher Beach, Dr. George Carpenter, Mr. D'Arcy Power, Dr. Humphrey Rolleston, Dr. George Shuttleworth, Mr. G. Sutherland, Mr. Alfred Tubby, Dr. Dawson Williams, and a number of other well-known medical men.

The Hon. Secretary of the Imperial War Fund has issued information as to the various funds which are assisting the soldiers who have taken part in the war in South Africa, and their widows, wives, and children. The principal associations are the Royal Patriotic Fund, the Soldiers and Sailors Families' Association, the Mansion House Fund, Lloyd's Patriotic Fund, the *Daily Telegraph's* Widows and Orphans' Fund, and the *Daily Mail* Fund. The Imperial War Fund can do nothing further at present for want of means. It has paid out over £8,000 to widows, orphans, and dependent relatives of soldiers.

The South African Hospitals Commission are carrying on their investigations, and have taken a large amount of evidence. Some of the men now at Netley have been interviewed, so has Mr. Burdett-Coutts, and numerous responsible eye-witnesses have told their tale to the Commissioners. The investigations cover a very large field, and every point, from the supply of milk to individual hospitals to the general transport arrangements, appear to be under consideration. On one point the witnesses seem to be unanimous, and testify to the indefatigable work of all members of the Army Medical Corps.

Among the various congresses which have made the Exposition Universelle in Paris the occasion for holding their meeting in that city one of the most interesting is that on Medical Ethics or Deontology, which met last week to discuss the many problems connected with the public relations of medical men. Among other subjects treated were those of Medical Men and Friendly Societies, International Reciprocity Between Medical Men, Hospital Abuse, Illegal Practice, and Medical Unions. In regard to some of these it soon became evident that the conditions of practice abroad were rather different from those in England; but it became equally clear that on many points the same ethical questions are constantly cropping up in every part of the world.

It is pleasing to record that the cyclists are doing one great kindness to the beast they are so rapidly supplanting, in trying to put a stop to the dangerous practice of throwing broken glass into the road, a practice which has been the cause of lameness and much misery to many a good horse. Even so far back as the year 1844 an Act of Parliament gave power to magistrates to punish people for so doing. But Acts of Parliament are of but little use unless they are enforced, and to enforce them often requires the action of some corporation rather than of many individuals. When cyclists, however, take a matter up they are apt to show themselves men of power, and so this old Act, which so long as it affected horses only did but little, is now showing renewed vitality under the stimulating auspices of the various cycle and pneumatic tyre-using associations. If they can put a stop to the senseless practice of throwing broken bottles out on to the roadway they will do a great public good.

The occasion of the centenary of the Royal College of Surgeons must make many people ask whether this rich and important corporation is doing all it might—one may almost say all that it should—do to promote and extend medical and surgical knowledge. With its wonderful museum, with its library, with its lecture-room, and all the appliances which it has at its disposal for advanced teaching something more might surely be expected from it than the few set lectures which are from time to time given to small audiences with a view rather to publication than to immediate interest. One cannot but feel that all should not be ended when a man has received his diploma from his college, and that, if its resources were utilised to the full, a member of the college might very properly expect to receive from it a great deal of that post-graduate teaching of which he so often feels the want as years go by. Diploma-giving should not be the highest functions of such a college. To teach its members and to guide and control their conduct should be well within its powers.

